# Which medical and social decision topics are important after early diagnosis of Alzheimer’s Disease from the perspectives of people with Alzheimer’s Disease, spouses and professionals?

**DOI:** 10.1186/s13104-016-1960-3

**Published:** 2016-03-08

**Authors:** Katharina Bronner, Robert Perneczky, Rose McCabe, Alexander Kurz, Johannes Hamann

**Affiliations:** Department of Psychiatry and Psychotherapy, Technische Universität München, Munich, Germany; University of Exeter Medical School, Exeter, UK; Neuroepidemiology and Ageing Research Unit, School of Public Health, Faculty of Medicine, The Imperial College of Science, Technology and Medicine, London, UK; West London Cognitive Disorders Treatment and Research Unit, West London Mental Health Trust, London, UK

**Keywords:** Alzheimer’s disease, Dementia, Qualitative research, Social decisions, Shared decision-making

## Abstract

**Background:**

The relevance of early decision making will rise with increasing availability of early detection of Alzheimer’s disease (AD) using brain imaging or biomarkers.

**Results:**

Five people with mild AD, six relatives and 13 healthcare professionals with experience in the management of AD were interviewed in a qualitative study regarding medical and social decision topics that emerge after early diagnosis of Alzheimer’s disease. Medical treatment, assistance in everyday life and legal issues emerged as the main decision topics after an early diagnosis of AD. People with AD mostly got in contact with the health and social care system through the initiative of their spouses. They were usually aware of their illness and most received antidementia drugs and/or behavioural interventions. Following diagnosis people with AD received support by their spouses. Healthcare professionals were aware of the risk of excessive demand on relatives due to supporting their family member with AD. In the opinion of healthcare professionals legal issues should be arranged in time before patients lose their decisional capacity. In addition, people with AD and spouses reported various coping strategies, in particular “carry on as normal” after diagnosis but mostly are reluctant to actively plan for future stages of the disease.

**Conclusions:**

Due to the common desire to “carry on as usual” after a diagnosis of AD, many people with AD and spouses may miss the opportunity to discuss and decide on important medical and social topics. A structured approach e.g. a decision aid might support people with AD and spouses in their decision making process and thereby preserve persons’ with AD autonomy before they lose the capacity in decision-making.

## Background

In recent years, major attempts have been made to establish the diagnosis of Alzheimer’s disease (AD) as early as possible to offer “timely access to information, advice, and support and access to a pathway of effective intervention and care from the time of diagnosis to end of life care” [1]. Thus, it is assumed that patients benefit from knowing about their disease and its prognosis early so that they can decide on their treatment and make plans for their future as long as they are still capable of doing so.

In that regard various studies have examined experiences of patients and family carers following an early diagnosis of AD. Investigators have indentified needs and preferences of people with early-stage AD e.g. participation in care planning and decision making [2] or disclosure vs. non-disclosure of the diagnosis [3]. Some studies addressed patients’ and relatives’ coping strategies e.g. holding on, compensating, fighting [4], subjective experience of dementia and its relevance for quality of life [5–8]. However, despite the above cited research, there are to our best knowledge no studies that have tried to determine *concrete decision topics* which are considered important by individuals who have received an early diagnosis of AD, their relatives and healthcare professionals and to display how these topics are actually dealt with.

The focus of the present research was therefore to explore important medical and social decision topics, which—if participation of the respective individual is desired—have to be addressed within a narrow time frame because persons with AD progressively lose their cognitive functions.

We believe this issue to be of growing (ethical) relevance not only due to the increasing availability of early detection of AD using brain imaging or using biomarkers but moreover, because still existing barriers in the diagnosis of AD, especially in primary care [9, 10] can be expected to be at least reduced in future years. Within a cross-sectional qualitative interview study, we aimed to identify medical and social topics which become relevant in the period following diagnosis of AD, for which a decision may eventually need to be made and which has implications for the life and wellbeing of the persons with AD. In addition, we aimed at describing how people with AD, spouses and professionals actually address these topics.

## Methods

### Participants

We used purposive sampling [11] to allow for maximum variation in the characteristics of professionals. Therefore we recruited professionals with various backgrounds including those involved in early diagnosis of AD, in counseling persons with early AD and in the management of all stages of dementia and in legal aspects of dementia care.

Patients and spouses of patients (we did not include dyads), who met the inclusion criteria, were identified in the memory clinic of the Department of Psychiatry at Technische Universität München. We aimed to recruit patients for whom the diagnosis had been established within the previous year or patients in whom diagnosis of AD had been made earlier but still had good cognitive functioning.

All patients had mild AD according to NINCDS-ADRDA criteria [12]. The diagnosis was confirmed by clinical and neuropsychological examination [Consortium to Establish a Registry in AD (CERAD)] Neuropsychological Assessment Battery [13], Mini-Mental-State Examination MMSE [14]. Additionally, some patients received 18F-fluorodeoxyglucose positron emission tomography (FDG-PET) imaging or cerebrospinal fluid (CSF) analysis for total-tau and Amyloid-β42 concentrations. Only patients with an MMSE score >24 were included to ensure that they were able to participate in the interviews. All relatives were spouses of persons with mild or moderate Alzheimer’s disease.

### Data collection

We chose a qualitative approach to get a better insight into the participants reasoning regarding the issue. The study was designed by a team of physicians and clinical psychologists who are familiar with the clinical problems associated with dementia. Interviews were conducted by a resident physician (KB) with previous research expertise in the field [15].

A topic guide for semi-structured interviews was developed by the research team and resulted in slightly different versions for professionals and for patients and relatives (see Table 1 for details). All interviews were conducted in face-to-face meetings and lasted 30–60 min. Data were collected at the memory clinic and at the professional’s workplace. All interviews were audio taped and transcribed verbatim.

**Table 1:** Guided Interview for professionals, patients and relatives

Decision topics	Interview with professionals	Interview with patients/relatives
Medical complaints of the patients, contact with the medical system, treatment	1. First of all I would like you to tell me some basics about your occupation What persons are generally contacting you? At what stages do clients contact you? Who does contact you (patients, relatives, other persons)?	1. First, please tell me for what reason you are here (at the department) and what is done with you? How do you get here? What problems have made you come here?
Time of diagnosis: disclosure, reactions	2. How does the process of diagnosis and disclosure of persons affected proceed? Reactions of persons affected Important issues Treatment Support	2. Now I would like to ask you about your diagnosis: At what time was it told to you? What have you heard about it? How was your reaction?
Main topics emerging after the early diagnosis of Alzheimer’s Disease	3. What issues are coming up when you are engaged with patients or family members? (Most frequent? Dramatic? Easy/difficult to resolve? 4. If you think of the beginning of Alzheimer’s Disease, what deficits regarding care and support of patients and relatives do you see?	3. What’s next? How will you deal with the disease? Are there any things that you think should be managed or planned? What kind of things? Are there any things that other persons or your relatives have told you that should be managed or planned? What kind of things?

### Data analysis

The transcripts were analyzed using content analysis, as described by Mayring, using the key steps of summarizing, explicating and structuring [16]. The data were reduced to the main statements, with repeated material being deleted. The material was then coded using categories emerging from the data. The analyses were documented and discussed by two researchers (KB, JH). To enhance validity, interview material was also presented and analyzed in a qualitative workshop (“Qualitative Werkstatt”) involving 16 scientists experienced in qualitative research. Here, the transcripts were coded independently by four groups of researchers who were not involved in the research project but were familiar with qualitative research methods. They discussed similarities and differences in codes afterwards. In this workshop a preliminary coding tree was developed, which was then applied to all of the transcripts and was modified during the coding process.

## Results

### Sample

A total of 24 individuals participated in the study: five people with AD (4 female), six relatives (three female) and 13 professionals (nine female). All relatives and all professionals who were approached agreed to participate in the study. Of nine people with AD approached, only one refused to participate because he did not want to discuss his medical complaints at that time. Three persons with AD were excluded because of limited cognitive ability: one person with AD scored below the predefined score of the MMSE and another two persons with AD were included but were later excluded due to considerable difficulties in understanding during the interview.

Persons’ with AD mean age was 65 years (SD ± 8.8), and the mean MMSE score was 25.5 (SD ± 1). All relatives were spouses of patients with AD. Professionals came from different work areas (three physicians, six social education workers, two professional legal guardians, one nurse specialising in palliative care, one private carer).

### Main decision topics emerging after early diagnosis of AD

The main decision topics identified by patients, relatives and professionals as important after an early diagnosis of AD were: (1) medical treatment, (2) support from family members and external help in everyday life and (3) legal issues. As many quotations of the participants did not refer to concrete decision topics, but addressed various aspects of coping with AD, we included a fourth category: (4) subjective coping with the illness of people with AD and their spouses. The main topics, sub-categories as well as verbatim quotations are displayed in Table 2.

**Table 2:** Main topics, their definition and verbatim examples from transcripts (P = Patient, A = Relative, E = Professional)

Topics	Statements
Medical treatment: All measures applied by healthcare professionals (including consultations, disclosure of test results and diagnosis, drug treatment etc)	
Getting in contact with the medical system	P2: “We have got many good physicians in our social environment, also psychiatrists, and my husband consulted them” P6: “…I didn’t want that, but my husband kept at it. He said: You have to do something!”
Referral to specialists	A1: “Our son immediately made an inquiry and she (patient) was sent to the memory clinic soon after” P3: “… and then we have seen a psychologist once in a while and he…. recommended your clinic” E1: “First position is normally the GP, then a medical specialist, then transferred here” E1: “There are usually relatives with the patient…That a patient gets in touch himself, does happen, but seldom” E3: “… it occurs that patients come by themselves. Primarily if they live alone…..but normally, if somebody lives in a partnership, relatives get in touch and contact us”
Diagnostic workup	P6: “Something is wrong with my cerebrospinal fluid. There are two things contained in, which pointed to Alzheimer’s Disease…”
Education and patients’ awareness of the disease	P4: “I know the diagnosis….I know, that it progresses slowly” P6: “It was written in a medical report, that it is suspected AD…. I wouldn’t like to come to an end like my sister and my mum, that I don’t know anything at all at the end” P 2: “This is still a mystery for me. My disease is a mystery, how this has happened…It meant, that I have to go to the psychiatric hospital, but what really takes place and what is the impact of the disease…?” E3: “Anyway above all they were educated about the medical things…..about the process of the disease” E9: “I think, that most people struggle pretty hard to have a basic understanding, to understand a bit better what it is all about…”
Drug treatment, behavioural interventions, study participation	P 6: “Conversations, read the newspaper, memory training, read newspaper articles and so on” A 3: “One day a doctor asked if we want to participate voluntarily in a pharmaceutical study” E 3: “They get Aricept or Exelon or the usual remedies. Additionally referral to a General Practitioner, treatment of depression…, even more regular appointments” E5: “They usually get pharmaceutical treatment…after the outpatient clinic…activating groups, daycare, care groups, memory training, even such a thing”
Assistance by family/external assistance and help: (Social) support provided by family members or external help	
Desired assistance	P6: “Anyway, I always refused outside help actually. Because I think, that husband and wife should be around for each other. And through this, my husband feels good, he is happy to do that” P4: “My family will care for me if I can’t do it” A1: “I’ve done everything myself. That works, because I’m at home. But this is not the case for everyone” A3: “Being the patient’s companion is in any case…..a full-time job, with the result that I can forget about my old job…..We discussed that at that time and our sons said that they are available anytime if needed and want to support their mum” A2: “But there wasn’t a problem for me, nursing and all such things. I’m very resourceful” A3: “I would like to have a Bosnian cleaner, if it would be possible” E8: “This is often the case: relatives promise their mum she never has to go in a retirement home. I think they can’t imagine the consequences if their mum develops severe dementia” E3: “Duties are arranged completely differently. And the wife has to completely start learning things from the beginning and complete things e.g., forms she has never done before. These are details, but it could become very difficult in individual cases” E2: “Well, it is all new at the beginning and it is important for relatives to build a social network” E9: “I think there are enough options to inform oneself and aids, initially, as long as it works well”
Housing situation	P 5: “…as long as I can stay in my flat” P5: “Yes, I was there (residential home)…, when it opened. I went there and looked at it” A3: “…and now I see my dedication to keep as active as possible, to be able to accompany her” E1: “Well, only seldom someone is so prepared or would like to be prepared or that he just knows he will go there (residential home). This is less common” E6: “The majority of the relatives would like to keep patients at home as long as possible” E8: “It is difficult for those who live alone…..The question actually is: Who is helping elderly people living alone with in the early stages of dementia?”
Legal issues: All issues relating to civil law (including legal capacity, regulations by law, last will, official guardianship, insurances etc)	
Legal issues	P3: “The two of us have done it, also the advance health care directive” A3: “Advance health care directive, health care proxy….and we have accordingly executed documents along with our sons…..the only thing missing is the last will…..Well, that remains to be done. That is on the list and we will do it as fast as possible” E 1: “The big issues of people with mild dementia are health care proxy, i.e., the legal regulations to be arranged” E 9: “In the early stages it is about clarification….and legal provision, power of attorney and as for me advance health care directive” E8: “The thing is mostly that another person such as a legal guardian or an authorised person should represent the dementia patient’s desires….People can’t imagine at the beginning that it might come to this….I think it would be easier for relatives if professionals tell them how serious it becomes if someone is in late stages of dementia” E10: “A big issue is car driving. The problem is that they still drive their car and we have to fight that they give up driving….and we have to call the police”
Coping with illness: Topics were categorized here when they affected the individual person and his or her coping with the disease. The issue car driving for example was categorized into “civil rights” when there was a debate about driving capacities and insurance issues but categorized under “subjective response” when the ability to drive a car was mentioned in the context of personal autonomy	
Patients’ affective reactions to their diagnosis and symptoms	P2: “This is a massive and terrible feeling for me” P4: “Relief, because I knew now I have got something” A 4:”Actually quite calm because the situation wasn’t bad yet” A3: “In my mind this was an essential shock for my wife. She is living with it, but she hasn’t really accepted the diagnosis until today” E3: “They are sinking into depression…..The person concerned, many say, doesn’t feel like doing anything, is retired, doesn’t want any contact” E1:”Certainly also anxiety, fright and sadness, but even so a piece of relief” E5: “Then clients don’t hear the diagnosis dementia too much; they hear rather that they have got depression”
Relatives’ difficulties of comprehension/handling with patients	P3:”And there isn’t comprehension that could also help me…” A3: “One of the essential points is the deficit in short term memory. Just to accept it as it is and for Christ’s sake not always spell it out…” A6: “It often causes trouble, if she asks me for the third time, then I don’t respond to her in a friendly way…” E1:” About coping with everyday life. This is the most difficult….how can I handle it, the disease and the deficits, which happen every day and everyday life constitutes a challenge”
Patients’ autonomy vs. paternalism by relatives or professionals	P6: “Not even my children suspect it (that I have dementia)….But I will tell them, when I think it is right” P2: “My husband talks to the physicians and is more familiar with that. I was present during the consultation, but I wasn’t able to participate actively. I’m sitting nearby, half-involved. I haven’t much knowledge, which my husband and the physicians have” P3: “Subsequently, my wife always checked the phone, to see with whom I have spoken” A5: “Then we participated in a study….and then it happened with the study and we said: Yes, we participate!” E10: “The issue is: I want to maintain my autonomy. I don’t want to be patronised” E3: “Duties are arranged completely different” E10: “…the relationship constellation gets mixed up totally and about the whole story is: Now they decide about me!” E1: “…participates or could participate at that time, it was still possible” E3: “The issue of driving is a very difficult issue, primarily for men. You don’t have to stop driving immediately with getting the diagnosis, but you have to discuss it”
“Carry on as normal”	P5: “And I try to do everything possible, as long as I possibly can” P2: “My husband won’t work eternally, still a few years. This isn’t arranged yet” A 2: “Actually nothing. We take it as it comes and we make the best of it” A4: “So I didn’t worry at all (about the disease) in the beginning, because I thought it couldn’t get so bad” E1: “Many people have got a big longing for continuing with what had been important all their life…. What represented my life, what I have always done, I would like to keep doing it” E2: “…there is a time slot… for legal questions e.g., guardianship law, health care proxy or advance health care directive” E9: “There are some specialists reading literature and confronting me with questions about the latest (study) results and some are very well informed. But this is more of an exception” E9: “…I think you have to accept, if someone doesn’t want to deal with it so early”

#### Medical treatment

For most people with AD, spouses or primary care physicians had initiated the referral to a medical specialist or to the memory clinic. Only rarely people with AD had made the decision themselves to get in contact with the health and social care system (“There are usually relatives with the patient…That a patient gets in touch himself, does happen, but seldom.”).

All persons with AD had received some kind of medical treatment, for example referral to an outpatient day clinic, medication or participation in a clinical trial. However, none of them mentioned drug treatment unless directly asked for it, although all had been prescribed antidementia drugs. On the other hand, they spontaneously provided information about other medical treatment and behavioural interventions like memory training. Spouses were better informed about the medical treatment of the persons with AD and provided more detailed answers. Almost all professionals spoke about drug treatment of AD, but also about other specific therapeutic options such as occupational therapy, physiotherapy or memory training. They also mentioned the possibility of participating in self-help and other supportive groups.

#### Assistance by family members and external help

After diagnosis, and/or because of disease progression, all people with AD received more support from their relatives than before. Some people with AD took this for granted (“Anyway, I always refused outside help actually. Because I think, that husband and wife should be around for each other. And through this, my husband feels good, he is happy to do that.”) while others acknowledged this as a special effort. Many spouses considered it as their duty to accept this challenge and take the time to support their family members. Two spouses even quit work.

As an additional result, the relationship between spouses changed. The diagnosis was a difficult adjustment effort on both sides, when certain duties were taken over by family members that were previously the responsibility of the affected person. None of the people with AD wished assistance from individuals outside the family context. They assumed that their relatives were responsible for this presently and in the future. In single cases, spouses expressed the need for more external assistance and support after diagnosis. However, most did not require assistance and did not seek and arrange for external help (“But there wasn’t a problem for me, nursing and all such things. I’m very resourceful.”).

Contrary to people with AD and spouses, nearly all professionals saw the risk that the relatives may be overburdened by making promise for future support without considering the further development of AD. Professionals thought it necessary for relatives to obtain external assistance for relief and to extend their social network to help support the person with AD. In their view, external assistance is available but needs to be actively sought by people with AD and relatives.

Similarly, the issue of making provisions for future nursing home admission was not of imminent importance for people with AD. Patients’ statements suggested that they wished to stay in their familiar housing situation as long as possible (“…as long as I can stay in my flat.”). Only one person with AD had collected information about old persons’ homes and had already visited one. For spouses, this issue was also not important, because they anticipated caring fully for the person with AD in the future. Professionals reported that this issue is often repressed or ignored for a long time. They also mentioned that difficulties are likely to arise when people with AD live alone and have no family to look after them or only have a limited support network.

#### Legal issues

Most professionals mentioned legal issues that emerge after a diagnosis of AD (“In the early stages it is about clarification…. and legal provision, power of attorney and as for me advance health care directive.”). These include legal matters of any kind that may be affected by the progress of dementia, e.g. making a will or providing advance health care directives.

Overall this issue was deemed very important by professionals, because they knew from personal experience the huge difficulties, in case these issues were not addressed when patients still have decisional capacity. In particular, professionals who were in contact with patients in advanced stages of AD emphasized the importance of making timely decisions, while people with AD could still play an active role.

Only two out of five people with AD talked about legal issues. Three spouses did not address this issue. The remaining three, however, dealt with this matter and took concrete measures.

Here, an important issue was the possible revocation of the driving licence of persons with AD when they drive their car although they should not because of their mental condition. One of the professionals reported that she hinders persons with AD from driving any longer. If necessary the police was even called.

#### Additional data on “Coping with illness”

Persons with AD reported different and sometimes mixed reactions to a diagnosis of AD, from fright, sorrow and fear to relief. Relatives’ reports of how persons with AD reacted were equally diverse. Confidence, hope and calmness were also described. Professionals’ reports how affected persons and their relatives might react, were more extensive than those of person’s with AD and relatives. They frequently mentioned fear, shock, despair, uncertainty and depression, but also relief.

Relatives’ difficulties in comprehending/handling patients and the disease were mentioned by persons with AD and spouses (“It often causes trouble, if she asks me for the third time, then I don’t respond to her in a friendly way…..”). Symptoms of dementia added to problems in everyday life. Professionals reported relatives’ difficulties dealing with persons with AD because of carers’ lack of understanding about how the symptoms of AD affect everyday life. For many persons with AD, the issue of autonomy becomes evident after diagnosis. An important example is the decision when and whom to inform about their diagnosis (“Not even my children suspect it…. But I will tell them, when I think it is right.”).

The autonomy of persons with AD is often restricted by relatives’ paternalism. Three persons with AD reported that relatives often took over negotiating when they visited the doctor together (“I was present during the consultation, but I wasn’t able to participate actively.”). Persons with AD felt only physically present and weren’t properly involved in the consultation by relatives and doctors.

Distribution of power changed within the family. The relatives’ paternalism also became clear in the wording of some relatives when they used the word “we” instead of “he” or “she”, if they reported on affairs concerning only the person with AD.

Relatives’ paternalism leads to conflicts when persons with AD feel dominated or controlled. Professionals looked upon loss of autonomy as a problem, especially when relatives dominate persons with AD in the early stages of AD, although they were still capable of making decisions at that time. Driving was a specific point of conflict in this respect. Giving up driving was a huge step with respect to the persons’ with AD independence. Sometimes, driving licence authorities had to be consulted to resolve this conflict.

Many persons with AD and relatives tend to carry on their daily lives as before even if competence for everyday life is declining. Moreover, persons with AD and relatives often saw no need for further planning (“Actually nothing. We take it as it comes and we make the best of it.”), as long as the symptoms were mild. Professionals, however, supported timely discussions about the future.

There were some hints in the interviews as to why persons with AD and relatives may abstain from actively planning for the future. Some professionals assumed a lack of motivation and interest for some persons with AD but not for all. They deny the reality, trivialize and displace their diagnosis. Furthermore, there is not only insufficient communication between persons with AD and their relatives, but also between persons with AD and GPs. They, however, acknowledge that patients have the right not to know or not to confront their disease.

## Discussion

Our data show several decision topics emerging after early diagnosis of Alzheimers’ disease. Many of these topics coincide with recommendations from guidelines [17] or self help groups, such as the Alzheimer’s Society or Alzheimer’s Association. These range from medical issues (treatment), social issues (support, housing), legal issues (health care proxy) and very personal issues (lifestyle). However, people with AD, relatives and professionals hold different views on how these topics should be prioritised in the time period after an early diagnosis of AD.

All persons with AD received drug treatment and behavioral interventions, which is not surprising given that they were recruited in a specialized memory clinic. Relatives and professionals also acknowledge the meaningfulness of the initiation of both drug treatment and behavioural interventions after diagnosis of AD. Thus, regarding these decision topics there is agreement of our results with general recommendations of guidelines, e.g. EFNS guideline [18]. In addition, many of the relatives and professionals also mentioned participation in a clinical trial as a possibility to improve outcomes. This is of interest since clinical trials do not aim at improving the individual’s condition but rather to produce generalizable knowledge.

There was more heterogeneity in the participants’ quotes regarding support from family members or from external help. People with AD saw it as their relatives’ responsibility to care for them and also the relatives felt committed to care for their family members. This is in line with results from a previous review [19], in which the main caregiver was predominantly a member of the family. Professionals had a completely different view, probably guided by their experiences with people in advanced stages of dementia. They strongly recommended that relatives early start planning an extension of their social network and external support to be supported in caring for the patient (e.g. nursing home) [20].

For legal matters, professionals again emphasized the necessity of timely planning and action as long as people with AD were still capable of making decisions. Many persons with AD as well as relatives, however, tended to postpone these decisions because they saw no immediate need to make them soon after the diagnosis. These findings appear to be consistent with research on advance planning by persons with dementia who do not realize the importance of planning until it was too late to discuss [21]. GPs may have problems to give people with dementia in early stages the understanding of the need of planning their end-of-life-care and see the future lack of person’s with AD decision-making capacity as a specific barrier to initiate advance planning [22].

The additional theme of “coping with the illness” may help to explain these differences between the attitudes of persons with AD, relatives and professionals towards timely planning of psychosocial decisions.

Obviously, many persons with AD are somehow petrified when they receive a diagnosis of AD, which then switches to a mindset of “carry on as normal”. Here we are in line with recent research that has even shown that persons’ with AD needs “to come to terms with the disease and maintaining normality” appeared to be very important. People diagnosed with dementia try to continue their daily routine as well as in any way possible [23]. Neither of these states of mind seems to facilitate “advance care planning”. A similar pattern might be true for the relatives, who might often underestimate the impact of advanced stages of dementia on everyday life [21].

When relatives take over the persons’ with AD responsibilities, it may be their intention to support persons with AD. However, they may consciously or unconsciously hinder important decision-making and long-term planning by persons with AD while they still have decisional capacity and thereby override the patient’s will in the long run [24].

Therefore we suggest that the persons’ with AD (and relatives’) emotional response, the avoidance (or denial) of the further course of the illness as well as the relatives’ and probably physicians’ paternalism are the main factors impeding advance care planning after early diagnosis of AD.

What makes these results increasingly relevant, however, is the fact that the number of people with AD getting their diagnosis early stages will steadily rise due to the increasing availability of early detection of AD using brain imaging or biomarkers. In addition, there are increasing opportunities for detection of dementia even in preclinical stages at genomics and biotechnology companies (e.g. 23 and Me). Finally, GPs might better diagnose early stages of AD.

## Limitations

By recruiting patients and relatives exclusively from a specialized memory clinic, this was a selected sample of participants, mostly persons who were concerned about minimal cognitive deficits and therefore actively seeking help.

In addition, it was not clear, whether patients did not remember certain topics such as diagnostic education due to ongoing memory loss/denial or whether full disclosure about the diagnosis had not taken place. In addition, our study has a limited sample size of a small number of patients and a selected unique sample of relatives (spouses only).

Finally, there may be differences in decision topics depending on which stage of AD exists and our results may therefore not be valid for persons with preclinical stages of AD without symptoms.

### Implications for clinical practice and further research

People with AD, relatives, professionals and guidelines propose many topics that should be addressed early in the course of AD before cognitive decline hinders patient participation in these decisions [25]. However, emotional reactions as well as restriction of persons’ with AD autonomy, may serve as barriers to timely addressing these topics. This raises the question of whether it would be better to actively strive for these decisions to be made or to respect procrastination and avoidance of these topics by persons with AD and relatives [26]. We would suggest that any intervention addressing this issue should directly approach patients (rather than only relatives) to support their autonomy in making decisions. In addition, it must deal with persons’ with AD emotional responses (ranging from shock to denial) consistent with past research [27].

In addition to existing dyadic interventions [26] the development of decision aids for people with AD could be a promising approach. Decision aids are already used for other somatic diseases (e.g. diabetes, heart disease) and have been shown to increase patients’ knowledge and their participation in decision-making. For Alzheimer’s disease, currently only one decision aid exists for persons with AD focusing on taking medication or not [28]. It would be of interest to explore whether a decision aid makes a difference in making more explicit decisions and whether they are made earlier. The REVEAL study [29] has shown that persons who were informed that they were at higher risk of developing AD (according to genetic testing for apolipoprotein E) were motivated to reduce risk by engaging in health relevant behaviors (e.g. medication/vitamins, diet or exercise) even if effectiveness of such activity is uncertain. Although relevant decision topics for persons at risk for AD may significantly differ from those of persons with early stage AD a decision aid may also lead people with early AD to take action earlier e.g. make timely decisions.
